# Direct Ex-Vivo Evaluation of Pneumococcal Specific T-Cells in Healthy Adults

**DOI:** 10.1371/journal.pone.0025367

**Published:** 2011-10-24

**Authors:** Aamir Aslam, Helen Chapel, Graham Ogg

**Affiliations:** 1 MRC Human Immunology Unit, Oxford NIHR Biomedical Research Centre, Weatherall Institute of Molecular Medicine, University of Oxford, Oxford, United Kingdom; 2 Clinical Immunology Unit, Nuffield Department of Medicine, University of Oxford, Oxford, United Kingdom; Health Protection Agency, United Kingdom

## Abstract

*Streptococcus pneumoniae* is an encapsulated bacterium that causes significant global morbidity and mortality. The nasopharynxes of children are believed to be the natural reservoir of pneumococcus and by adulthood nasopharyngeal carriage is infrequent; such infrequency may be due to demonstrable pneumococcal specific T and B-cell responses. HLA Class 2 tetrameric complexes have been used to characterise antigen specific T-cell responses in a variety of models of infection. We therefore sought to determine the frequency and phenotype of pneumococcal specific T-cells, using a novel HLA-DRB1*1501 tetramer complex incorporating a recently defined T-cell epitope derived from the conserved pneumococcal serine/threonine kinase (StkP). We were able to detect direct *ex-vivo* StkP_446–60_-tetramer binding in HLA-DRB1*1501 adults. These StkP_446–60_-tetramer binding T-cells had increased CD38 expression and were enriched in CCR7- CD45RA+ expression indicating recent and on-going activation and differentiation. Furthermore, these StkP_446–60_-tetramer binding T-cells demonstrated rapid effector function by secreting interferon-gamma on stimulation with recombinant StkP. This is the first study to directly enumerate and characterise pneumococcal specific T-cells using HLA class 2 tetrameric complexes. We found that *ex-vivo* pneumococcal-specific T cells were detectable in healthy adults and that they were enriched with cell surface markers associated with recent antigen exposure and later stages of antigen-driven differentiation. It is likely that these activated pneumococcal specific T-cells reflect recent immunostimulatory pneumococcal exposure in the nasopharynx and it is possible that they may be preventing subsequent colonisation and disease.

## Introduction

*Streptococcus _neumonia* (pneumococcus) is an extracellular bacterium that causes significant mortality and morbidity globally [1]. Young children are often nasally colonised and also have the highest incidence of pneumococcal infections. However with time, the rate of colonisation and infection falls and by late childhood the prevalence of nasal colonisation reaches a low-point – a state that persists into adulthood, although the incidence of pneumococcal infection increases in the elderly despite their maintaining relatively low rates of colonisation [2], [3], [4]. Pneumococcal exposure can lead to the generation of both B-cell and T-cell immune responses to polysaccharide and protein antigens [5], [6], [7], and although anti-capsular antibody responses generated by vaccination in children can prevent subsequent colonisation, the natural acquisition of immunity to pneumococcus precedes detectable rises in anticapsular antibody responses [8]. Furthermore, in adults the possession of high titre anti-capsular antibody responses does not necessarily protect against pneumococcal disease in selected patients [9]. T-cells can play an important role in the development and maintenance of class switched antibody responses, although T-cell independent B cell class switching can also occur. Indeed, anti-pneumococcal protein antibody responses are T-cell dependant [10] and T-cell responses, as expected, are detectable in adults and children to both whole pneumococcus and pneumococcal proteins and peptides; these have been demonstrated by measuring T-cell proliferation and cytokine secretion [6], [7], [8]. In addition to influencing antibody production by B-cells, T-cells can activate cell mediated immunity via the secretion of IL-17, IL-22 and IFN-gamma. It is likely that these responses are important in clearing mucosal colonisation in children and maintaining protective immunity in adults [11], [12]. Unlike children, young adults are rarely colonised with pneumococcus and have a relatively low incidence of pneumococcal infection. It is possible that pneumococcal specific T-cell immunity is contributing to this and we therefore sought to evaluate direct ex-vivo pneumococcal T-cells in healthy adults. Having previously defined an HLA-DRB1*1501 restricted MHC Class 2 epitope within StkP, we used StkP-HLA-DRB1*1501 tetrameric complexes to enumerate pneumococcal specific T-cells directly ex-vivo from healthy adults and to characterise these cells further in terms of maturity and activation status [12]. We found that pneumococcal specific T-cells were detectable in most healthy adults. Furthermore, these T-cells have increased expression of CD38, suggesting that they have been recently activated.

## Results

### Identifying pneumococcal specific T-cells

**Figure 1:**
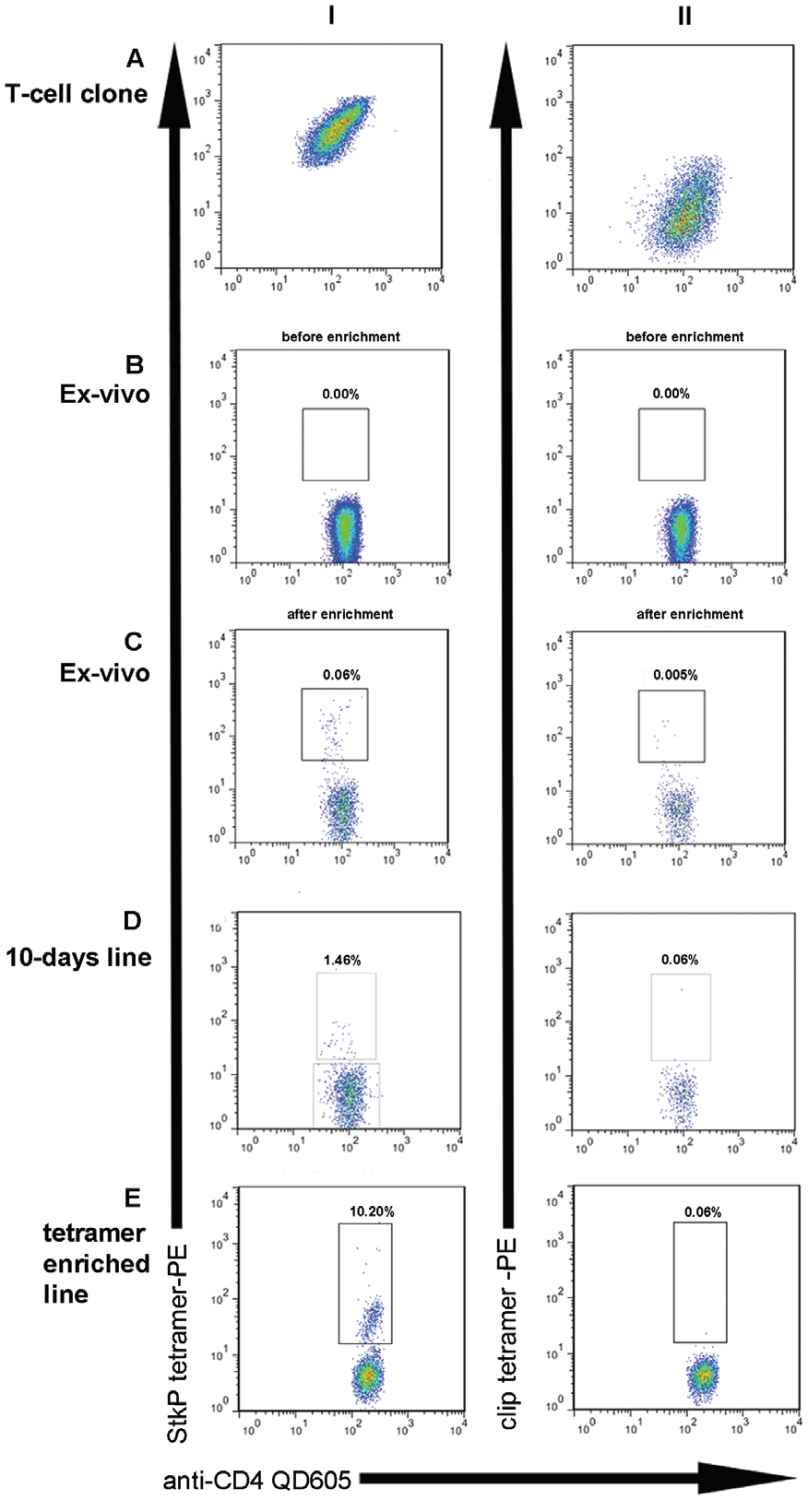
Identifying pneumococcal specific T-cells with a Class 2 tetrameric complex. Dot plots showing tetramer binding of CD4+T-cells from various cellular sources. Column I shows binding of CD4+ T-cells to the StkP_446–60_-HLA-DRB1*1501 tetrameric complexes and column II indicates staining with a control CLIP DRB1*1501 tetramer. StkP_446–60_-tetramer and control tetramer binding of a known StkP_446–60_-specific IFN –gamma secreting T cell clone from HV1 is shown in A. As StkP_446–60_-tetramer staining of *ex-vivo* samples (B) was not detectable in HV2, anti-PE magnetic beads were used to enrich with consequent detection of StkP_446–60_-tetramer binding (C). StkP_446–60_-tetramer binding CD4+ T-cells were also identified after 10 days expansion of HV2 PBMC with StkP_446–60_ peptide (D). This StkP_446–60_-tetramer binding population was sorted by flow cytometry and further expanded *in vitro* for 2 weeks with a subsequent further enrichment of StkP_446–60_-tetramer binding cells (E).

PBMC and derived T-cell clones and lines were derived from 10 healthy volunteers (HV1-10), all of whom expressed HLA-DRB1501. The HLA-DRB1*1501-StkP tetramer was able to bind to a pneumococcal specific IFN-gamma secreting T cell clone from HV1 (figure 1A); this clone had been generated by its ability to secrete IFN-gamma in response to the StkP HLA-DRB1*1501 restricted epitope QSFQISNYVGRKSSD (StkP_446–60_). Background non-specific tetramer staining was determined using the HLA-DRB1*1501-CLIP tetramer which contains the CLIP peptide (PVSKMRMATPLLMQA), that associates with HLA- Class 2 molecules during antigen processing. We next determined whether we could detect pneumococcal specific T-cells in healthy HLA-DRB1*1501 expressing adults and, as shown in figure 1b, the *ex-vivo* frequency was undetectable using PBMC from HV2. As ex-vivo epitope specific T-cell responses are often found at low frequencies, we enriched StkP_446–60_ tetramer binding cells in HV2 using anti-PE magnetic beads as has been done previously [13]. By determining the absolute CD4 T-cell count, we were able to calculate the percentage StkP_446–60_-tetramer binding and were able to show detectable ex-vivo StkP CD4+ T-cell responses following enrichment (figure 1C).

### Proliferative and effector capacity of tetramer binding cells

**Figure 2:**
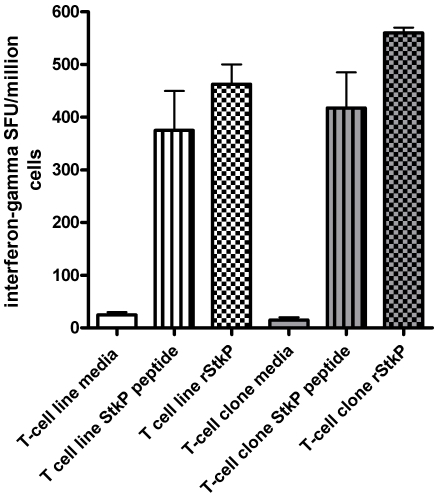
StkP_446–60_-HLA-DRB1*1501-tetramer binding T-cells secrete IFN-gamma. A tetramer binding T-cell line (unfilled colums) and a separate T-cell clone from HV2 were stimulated with StkP_446–60_ peptide (10 micromolar final concentration-vertical lines), recombinant StkP protein (10 ug/ml final concentration-chequered) or media (control-no pattern) for 16 hours. Interferon gamma secreting cells were enumerated by the ELISPot assay.

After expanding StkP-specific T-cells further, by incubating PBMC from HV2 with StkP_446–60_ peptide for 10-days, the proportions of StkP_446–60_-tetramer binding T-cells were increased (figure 1D), which suggests that tetramer binding cells are able to proliferate *in vitro*. Approximately one thousand of these tetramer binding cells were sorted by flow cytometry and, after further rounds of expansion, there was a marked increase in the frequency and absolute numbers (>10^7^ CD4 T-cells) of these StkP_446–60_-tetramer-binding CD4+ T-cells (figure 1E). This confirms that the tetramer binding CD4+ T-cells are capable of substantial proliferation in response to stimulation. In addition to their proliferative capacity, StkP_446–60_-tetramer-binding CD4+ T-cell lines and clones were also able to secrete interferon-gamma. A StkP_446–60_-tetramer-binding CD4+ T-cell line and clone were stimulated with both StkP_446–60_ peptide and recombinant StkP and interferon gamma secretion was demonstrated using the ELISPot assay (figure 2).

### Frequency and phenotype of pneumococcal specific T-cells

**Figure 3:**
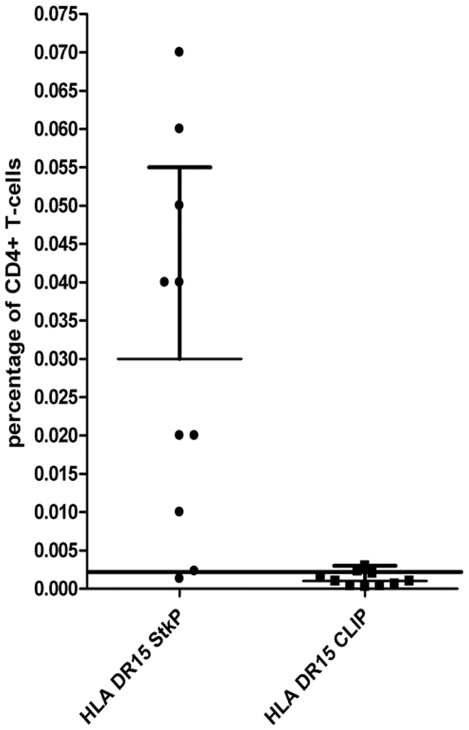
Frequency of Pneumococcal specific T-cells. The percentage of StkP_446–60_-tetramer and the control CLIP-tetramer binding was calculated in 10 healthy HLA-DRB1*1501 adults. The limit of detection (mean of the CLIP-tetramer binding +2SD) of specific StkP_446–60_-tetramer binding after magnetic-bead enrichment is 0.002% HV1-8 had detectable tetramer binding-i.e.frequencies greater than the limit of detection.

**Figure 4:**
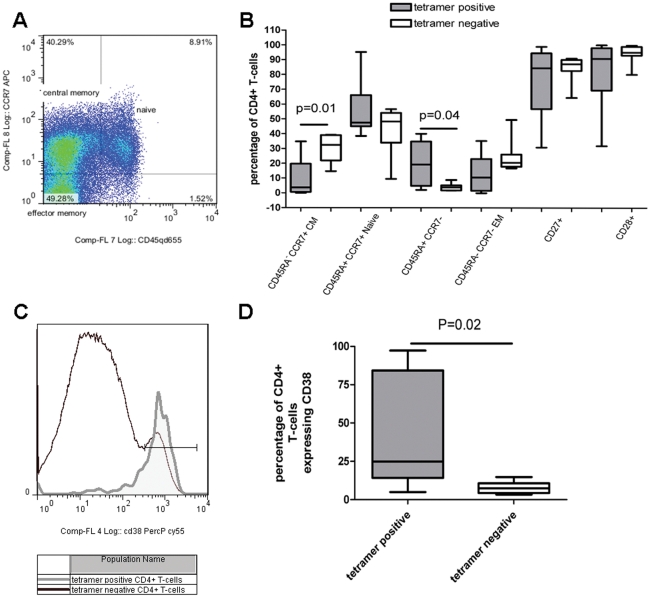
Phenotype of Pneumococcal specific T-cells. The phenotype of tetramer binding T-cells HV1-8, who had detectable tetramer binding was evaluated using concomitant staining of T-cell surface markers. T-cell memory subsets were identified on the basis of CCR7, CD45RA, CD27 and CD28 surface expression, (an example of which is shown in A). The frequencies of central memory (CM), effector memory, naive and mature CD45RA+ CCR7- subsets were determined in the StkP_446–60_-tetramer binding and StkP_446–60_-tetramer-negative populations in 8 healthy adults (B). CD38 expression was also determined in the StkP_446–60_-tetramer binding and the StkP_446–60_-tetramer -negative CD4+ T-cells (shown in HV3 in C, grey outline is tetramer positive cells and black outline is tetramer negative CD4+ T-cells). D shows CD38 expression in StkP_446–60_-tetramer positive (grey filled column) and negative (unfilled) cells from 8 healthy adults with a significant difference in the mean expression (Wilcoxon signed rank test, p = 0.02).

Using the StkP_446–60_-tetramer to enumerate the direct ex-vivo frequency of StkP-specific CD4+ T-cells, we found that 8 out of 10 healthy DRB1*1501 expressing adults had detectable responses (figure 3), with staining similar to that seen in HV2 after enrichment (figure 1C). Concomitant staining of cell surface proteins was used to determine the differentiation and activation status of these pneumococcal specific cells in these 8 healthy adults. The expression of CCR7 and CD45RA allows for the categorisation of T-cells into central memory (CM), effector memory (EM), naive and a mature CCR7 negative, CD45RA positive subset (figure 4) [14], [15]. The proportions of StkP_446–60_-tetramer binding CD4+ T-cells were enriched within CD45RA^+^ CCR7^−^ CD4+ T-cells and consequently reduced in CD45RA-CCR7+ CM CD4+ T-cells (figure 4, p = 0.04 and p = 0.01, respectively). There was no significant difference when comparing the StkP_446–60_-tetramer binding frequency of the other T-cell subsets to the tetramer negative population of T-cells, nor was there any difference when using the T-cell markers of differentiation, CD27 and CD28 (figure 4). Interestingly, using the expression of CD38 as a marker of T-cell activation, there was a higher frequency of CD38 expression in the StkP_446–60_-tetramer binding cells than in the tetramer negative population (figure 4).

## Discussion

This is the first report to use HLA-class 2 tetramers for the direct ex-vivo enumeration and characterisation of pneumococcal specific T-cells in healthy individuals. We have shown that 8 out of 10 HLA DRB1*1501 healthy adults have detectable StkP_446–60_-tetramer binding CD4+ T-cells with a range in frequency (0.01–0.07%) similar to frequencies observed in other models of exposure to viral and intra-cellular bacterial infections as well as following desensitisation therapy in allergic disease [16], [17], [18], [19]. HLA-class 2 tetramers identify CD4+ T-cells irrespective of the T-cells ability to proliferate or secrete cytokine. The increased frequency of StkP_446–60_-tetramer binding in StkP stimulated lines after 10 days expansion, as well as in StkP_446–60_-tetramer sorted and subsequently expanded T-cell lines, indicates that these StkP_446–60_-tetramer binding CD4 T-cells were able to proliferate *in vitro*. Furthermore both StkP_446–60_-tetramer binding CD4 T-cell lines and clones secreted IFN-gamma in response to the StkP_446–60_ peptide and recombinant StkP. Indeed, pneumococcal specific interferon-gamma responses have been detected in adenoidal tissue from children [20]. Thus, StkP_446–60_-tetramer binding cells CD4+ T-cells have rapid effector function in terms of interferon gamma secretion and proliferation.

The identification of antigen specific T-cells by HLA-class 2-tetramers allows the direct characterisation of *ex-vivo* T-cells without altering their phenotype as occurs when using antigen-based activation assays. We determined the expression of cell surface markers, CD45RA, CCR7, CD27 and CD28 that have been used to categorise CD4+ T-cells into distinct subsets. There is evidence that emergence of these distinct subsets of antigen experienced cells follows a progressive differentiation model. Accordingly, antigen experienced cells that express CCR7, CD27 and CD28 traffic to lymphoid tissue and are enriched with IL-2 secreting cells and represent early-differentiated cells. In contrast, as antigen experienced cells progress through sequential rounds of cell division, a substantial proportion will progressively lose CCR7, CD28 and CD27, with the eventual re-expression of CD45RA marking terminally differentiated cells [14], [15]. The loss of CCR7 allows for these late-differentiated cells to home to inflamed peripheral tissue where they can directly participate in the effector immune response to infectious agents. Our demonstration that HLA-class 2 tetramer binding cells are enriched in CCR7- CD45RA+ expression implies that they are terminally differentiated CD4+ T-cells. We also found that CD38 expression was increased on StkP_446–60_-tetramer binding T-cells. CD38 expression is an activation marker, suggesting that the StkP_446–60_-tetramer binding cells have recently encountered antigen. These CD38 positive cells readily produce cytokine and are more likely to apoptose, a feature that is typical of effector T-cells [21].

We know that the prevalence of pneumococcal nasopharyngeal colonisation is low in adults, ranging between 4–5.9%, although we did not directly determine if any our healthy volunteers were colonised [2], [22], [23]. It is therefore likely that the activation of the StkP_446–60_-tetramer binding T-cells reflects recent immunostimulatory exposure to pneumococci. It is possible that these activated T-cells may be more than markers of pneumococcal exposure, being directly involved in preventing such exposure from progression to sustained nasopharyngeal invasion. The loss of CCR7 expression - which favours trafficking of T-cells to peripheral tissue – is also consistent with a direct role for these StkP_446–60_-tetramer binding T-cells in pneumococcal immunity in the nasopharynx. Indeed, others have demonstrated that pneumococcal specific T-cell cytokine and proliferative responses are associated with nasopharyngeal pneumococcal sterility in children [24]. These subclinical, non (or very brief) colonising exposures may not only activate T-cells but may also drive their differentiation into the CD45RA re-expressing compartment.

We have used HLA-class 2 tetramers to show that healthy adults have detectable direct StkP_446–60_ responses to pneumococci. Our observation that these cells have been recently activated indicates that in healthy adults there is a dynamic T-cell response to presumed frequent exposures to pneumococci that do not progress to detectable nasopharyngeal colonisation or invasion. It would be of great interest to use Class 2 tetramers to enumerate and characterise the pneumococcal specific T-cell response during invasive pneumococcal infection. Given the increased risk of pneumococcal infection in the elderly, it would also be interesting to determine if there are any age-related changes in the frequency and phenotype of StkP_446–60_-tetramer binding cells, as occurs in other situations of persistent/ongoing exposure [16], [17].

## Materials and Methods

### Ethics Statement

The study was approved by Oxfordshire Research Ethics Committee (REC); all subjects gave informed consent.

### Subjects

Ten healthy adult volunteers (HV1-10) who expressed HLA-DRB1*1501 were recruited. Peripheral blood mononuclear cells (PBMCs) were separated from heparinized peripheral blood by density gradient using Lymphoprep (Nycomed, Roskilde, Denmark). PBMCs were then washed in RPMI supplemented with penicillin, streptomycin and l-glutamine (R0) and resuspended in RPMI with penicillin, streptomycin, l-glutamine and 10% fetal calf serum (FCS; R10).

### HLA Typing

All laboratory volunteers were HLA typed. Genomic DNA Puregene DNA isolation kit (Gentra Systems, USA) was used to isolate DNA from whole blood. HLA-A, -B, -C, DRB1, DRB3, DRB4, DRB5 and DQB1 specificities were determined using sequence specific primers by our in-house HLA typing service [25].

### Antigens

The StkP_446–60_ peptide QSFQISNYVGRKSSD was synthesized in-house in an automated synthesizer using 9-fluorenylmethoxycarbonyl chemistry and the purity of the peptides was determined to be greater than 90% by high-performance liquid chromatography (Gilson, Middleton, WI, USA) analysis [26], [27]. The purity of the peptide were confirmed by matrix-assisted laser desorption mass spectrometer on a Bruker Daltonics Ultraflex TOF/TOF mass spectrometer (Bruker, Billerica, MA, USA) [28].

### T-cell lines and clones

An interferon-gamma secreting StkP_446–60_ specific T-cell clone that had been previously generated was used to confirm specificity of the StkP_446–60_ tetramer [12]. StkP_446–60_ specific T-cells were expanded *in vitro* by incubating 4×10^6^ PBMC in 2mls R10* in 24-well plates (Corning) for 10 days with StkP_446–60_ peptide (final concentration 2 µM). Interleukin-2 was added on days 3 and 7 at a concentration of 100 units/ml. All cell lines were maintained at 37°C, in 5% CO2.

A StkP_446–60_ tetramer enriched T-cell line was generated by first sorting StkP_446–60_-tetramer positive CD4+ T-cells from a 10-days expanded T-cell line. Approximately 1000 of these cells were sorted into a single well of a round-bottomed 96-well plate using a MoFlo cell sorter. 100 µl of irradiated feeder cells – (1∶1∶1 of PBMC from 3 different individuals) at 1×10^6^ cells/ml in R10* containing IL-2 (100 IU/ml) and PHA (10 µg/ml) were added to the well.

### IFN-gamma ELISpot

ELISpot plates (Millipore Corp., Bedford, MA, USA) were coated with anti-human interferon (IFN)-γ overnight (Mabtech AB, Nacka, Sweden). The plates were washed six times with RPMI-1640 and blocked for 1 h with RPMI-1640 supplemented with 2 mM L-glutamine, 100 IU/ml penicillin and 100 µg/ml plus 10% human serum (R10*). 40,000 T-cell blasts from *in vitro* expanded T-cell lines or 1000 T-cell clones were added to each well to which StkP_446–60_ peptide (10 micromolar final concentration), recombinant StkP protein (10 ug/ml final concentration) or media (control) was added. Wells were set-up in duplicate. After overnight incubation at 37°C and 5% CO2, plates were washed ×6 in PBS-Tween 0.05% and incubated with 1 µg/ml of biotin-linked anti-IFN-γ (Mabtech AB) for 2 hours. After washing ×6 in PBS-Tween 0.05%, the plates were incubated for a further 1 hour with streptavidin-alkaline phosphatase (Mabtech AB). Spots were visualized using an alkaline phosphatase conjugate substrate kit (Biorad, Hercules, CA, USA) and enumerated using an automated ELISpot reader. Results were expressed as spot-forming cells per total number of cells after subtracting the background (cells alone).

### Tetramer staining

DRB1*1501 MHCII tetramer and hCLIP peptide HLA DRB1*1501 negative control tetramer were provided by the NIH Tetramer Core Facility at Emory University in Atlanta, GA, USA. DRB1*1501-PE tetramer was complexed to the StkP_446–60_ peptide QSFQISNYVGRKSSD, a previously defined HLA-DRB1*1501 restricted T-cell epitope [12] . Cell lines, T-cell clones and PBMC were incubated with 0.2 µg/ml HLA class II tetramer for 120 min at 37°C in R10* before staining with cell surface marker antibodies at room temperature for 20 minutes, including: anti-CD3 pacific orange, anti-CD4-quantum dot 605, anti-CD45 quantum dot 655, anti-CD27-FITC, LIVE/DEAD® Fixable Violet Dead Cell Stain (Invitrogen, Carlsbad, CA, USA), anti-CD14 pacific blue, anti-CD19 pacific blue (Biolegend, San Diego, CA, USA), anti-CCR7 Alexa647, anti-CD38 PercP Cy 5.5 and anti-CD28 PE Cy5 (*Becton*, *Dickinson* and Company, Franklin Lakes, NJ,USA). Stained cells were washed with phosphate-buffered saline (PBS) and fixed in 0•5% PBS/formaldehyde. Cells were acquired on a BD™ *LSR II* (BD) and analysed using FlowJo software (Tree Star, Inc. OR, USA). Gating strategy, singlet cells were first gated using FSC vs. FSC (area). Dead, CD14 and CD19 positive cells were then excluded using violet1 versus FSC.

Tetramer enrichment:

CD4+ T-cells were first enriched by negative selection using RosetteSep® (STEMCELL Technologies, France) from whole blood. The absolute CD4+ T-cell count was then determined using Trucount™ (BD) beads before staining with tetramer. The CD4+ T-cells were then incubated with anti-PE beads and positively selected using a magnet-based separation (Miltenyi Biotec, Germany). The tetramer enriched cells were then stained with antibodies directed to cell surface markers as mentioned above, before the entire sample was run through a BD™ *LSR II* (BD). The percentage of CD4 T-cells that were tetramer binding was calculated by dividing the total number of tetramer positive events by the total number of CD4+ T-cells that had been enumerated prior to enrichment.

### Statistics

Statistical tests were used to determine if the null hypothesis could be rejected at a probability of <0.05. Non-parametric statistical tests were used; Wilcoxon signed rank test using the statistical software package GraphPad Prism 4.
